# Four new species of the planthopper genus *Metanigrus* Tsaur, Yang & Wilson from China (Hemiptera, Fulgoromorpha, Meenoplidae)

**DOI:** 10.3897/zookeys.1024.62226

**Published:** 2021-03-16

**Authors:** Sha-Sha Lv, Thierry Bourgoin, Lin Yang, Xiang-Sheng Chen

**Affiliations:** 1 Institute of Entomology, Guizhou University, Guiyang, Guizhou 550025, China; 2 The Provincial Special Key Laboratory for Development and Utilization of Insect Resources of Guizhou, Guizhou University, Guiyang, Guizhou, 550025, China; 3 Institut Systématique, Evolution, Biodiversité, UMR 7205 MNHN-CNRS-Sorbonne Université-EPHE-Univ. Antilles, Museum National d’Histoire Naturelle, CP 50, 57 Rue Cuvier, F-75005, Paris, France

**Keywords:** Fulgoroidea, Oriental region, Meenoplinae, morphology, taxonomy

## Abstract

Four new species of the genus *Metanigrus* Tsaur, Yang & Wilson, 1986, *M. chromus* Lv & Chen, **sp. nov.**, *M. guttatus* Lv & Chen, **sp. nov.**, *M. gremius* Lv & Chen, **sp. nov.**, and *M. spinatus* Lv, Chen & Bourgoin, **sp. nov.** from China (Hubei, Guizhou and Yunnan), are described and illustrated, giving the genus six species in total. A key to all known species of *Metanigrus* is provided, as well as a map of their geographic distributions.

## Introduction

Meenoplidae is a small family of only 23 genera and 162 species representing, respectively, 0.9% and 1.2% of the generic and specific diversity the planthoppers (Hemiptera, Fulgoromorpha) (Bourgoin 2021). This family was established by Fieber (1872) with a single species from Greece. It is divided into two subfamilies which are easily separated by the claval veins (Pcu and CuA), which merge basally and with Pcu bearing a single row of sensory pits in Meenoplinae Fieber, 1872, while in Kermesiinae Kirkaldy, 1906 these veins merge distally in the clavus with a row of sensory pits on each side of Pcu (Wilson 1988; Hoch et al. 2014). Meenoplinae currently includes six genera and 59 species, and Kermesiinae has 17 genera and 103 species (Bourgoin 2021). Both lineages are mainly distributed in tropics and subtropics but absent from the Nearctic (Emeljanov 1984; Bourgoin 1997, 2021).

In China, meenoplid species have been less studied and are currently represented by eight genera: *Anigrus* and *Metanigrus* (Meenoplinae) and *Eponisia*, *Eponisiella*, *Kermesia*, *Nisia*, *Suva* and *Tyweponisia* (Kermesiinae), representing 23 species (Hu and Yang 1993; Liu and Qin 2020; Bourgoin 2021).

*Metanigrus* was established by Tsaur et al. (1986) based on the type species, *M. yami* Tsaur, Yang & Wilson, 1986 from Taiwan. Liu and Qin (2020) recently reported this species from Yunnan and described an additional new species, *M. rotundatus* Liu & Qin, 2020. Herein, four new species of the genus, *M. chromus* sp. nov., *M. guttatus* sp. nov., *M. gremius* sp. nov., and *M. spinatus* sp. nov. from China, are described and illustrated. There are now six species, all endemic to Chinese fauna. A key based on morphological characteristics to distinguish species is provided as well as a map of their geographic distributions.

## Materials and methods

Morphological terminology follows Bourgoin (1997) and Bourgoin et al. (2015) for the tegmina, Anufriev and Emeljanov (1988) for the wing, and Bourgoin (1987) for male genitalia. Body measurements are from apex of head to tip of tegmina. The metatibiotarsal formula LT-(T)/Mt1/Mt2 provides the number of spines on the side of the metatibia (LT) - on the apex of metatibia, eventually in two groups of internal (Ti) and external (Te) spines separated with a diasteman (Ti-Te) / on the apex of first metatarsomere (Mt1) / on the apex of second metatarsomere (Mt2). All measurements are in millimeters (mm). Dry specimens were used for descriptions and illustrations, external morphology was observed under a stereoscopic microscope, and characters were measured with an ocular micrometer. Habitus photographs were taken using a Nikon SMZ 25 digital camera and multiple layers were stacked using Helicon Focus 6. The genital segments were removed from the examined specimens and macerated in 10% NaOH. Illustrations were made using Leica MZ 12.5 stereomicroscope. The photographs and the illustrations were imported into Adobe Photoshop 6.0 for plate composition and labeling.

The type specimens examined are deposited in the Institute of Entomology, Guizhou University, Guiyang, Guizhou Province, China (**IEGU**).

## Taxonomy

### Metanigrus

Taxon classificationAnimaliaHemipteraMeenoplidae

Tsaur, Yang & Wilson, 1986

56B589B0-AEB1-5797-8002-149684343B26

Metanigrus Tsaur, Yang & Wilson, 1986: 108; Liu and Qin 2020: 29.

#### Type species.

*Metanigrus yami* Tsaur, Yang & Wilson, 1986, by original designation.

#### Diagnosis.

Small to medium-sized meenoplids. Vertex without median carina, with two posterolateral areolets at base not meeting medially. Frons without median carina. Postclypeus with median and lateral carinae. Pro- and mesonotum with median carina. Tegmina slender, with anterior and posterior margins subparallel, almost as wide at postnodal as prenodal area, MP3 two branched, five postnodal closed cells with C1 smaller than C2, C5 placed next to C3, C4 distally displaced after the nodal line, im, r-m and anterior two-thirds of margin with white areas. Hind wing with lateral margin notched at CuA1, CuP, and A2, more strongly at A1; A2 not reaching posterior margin. Metatibiotarsal formula: (3+5)-6-5. Male genitalia in lateral view, pygopher with internal sulcus, basolateral side of anal tube with a finger-like process, aedeagus acuminate, a membraneous lateral lobes with many scale-like productions; gonostyli divided into two parts, inner process curved apically, tapering into a process, outer process convex, inner margin with long setae.

#### Distribution.

China (Taiwan, Hubei, Guizhou, Yunnan) (Fig. 61).

### Key to species of *Metanigrus*

**Table:** 

1	Dorsolateral and middle parts of anal tube without finger-like process	**2**
–	Dorsolateral or middle parts of anal tube with finger-like process	**4**
2	Basolateral side of anal tube with a short finger-like process, outer process of gonostyli with a thumb-like process (Tsaur et al. 1986: fig. 15E)	***M. yami***
–	Basolateral side of anal tube with a long curved finger-like process, outer process of gonostyli without thumb-like process	**3**
3	Frons (Fig. 10) with two brownish-black patches on both sides, outer process of gonostyli (Figs 13, 16) bifurcated nearly equal at apex in lateral view	***M. chromus* sp. nov.**
–	Frons medially with a big, longitudinal, brown patch, outer process of gonostyli not bifurcated at apex in lateral view (Liu and Qin 2020: figs 3D, 4D)	***M. rotundatus***
4	Dorsolateral and middle parts of anal tube (Figs 35, 38) each with a finger-like process, outer process of gonostyli (Figs 35, 38) nearly square at apex in lateral view	***M. gremius* sp. nov.**
–	Dorsolateral or middle parts of anal tube with a finger-like process, outer process of gonostyli not nearly square at apex in lateral view	**5**
5	Distal part of tegmina (Fig. 22) with a large black patch; dorsolateral part of anal tube (Figs 24, 27) with a stout finger-like process	***M. guttatus* sp. nov.**
–	Distal part of tegmina (Fig. 44) without a large black patch; middle part of anal tube (Figs 46, 49) with a finger-like process	***M. spinatus* sp. nov.**

### Metanigrus chromus

Taxon classificationAnimaliaHemipteraMeenoplidae

Lv & Chen sp. nov.

181B5233-3490-5FBD-A149-18DEA1F5F202

http://zoobank.org/C529251B-F3CB-4B4F-B124-E664DA6F4813

Figs 1 , 2 , 9–19

#### Measurements.

Total length: male 3.90–4.31 mm (*N* = 18), female 4.31–4.75 mm (*N* = 31).

#### Diagnosis.

The salient features of the new species include: frons (Fig. 10) with two brownish-black patches on both sides; outer process of gonostyli (Figs 13, 16) differentiated into two processes nearly equal at apex in lateral view; only basolateral side of anal tube (Figs 13, 16) with a finger-like process.

#### Coloration.

**Figures 1–8.**
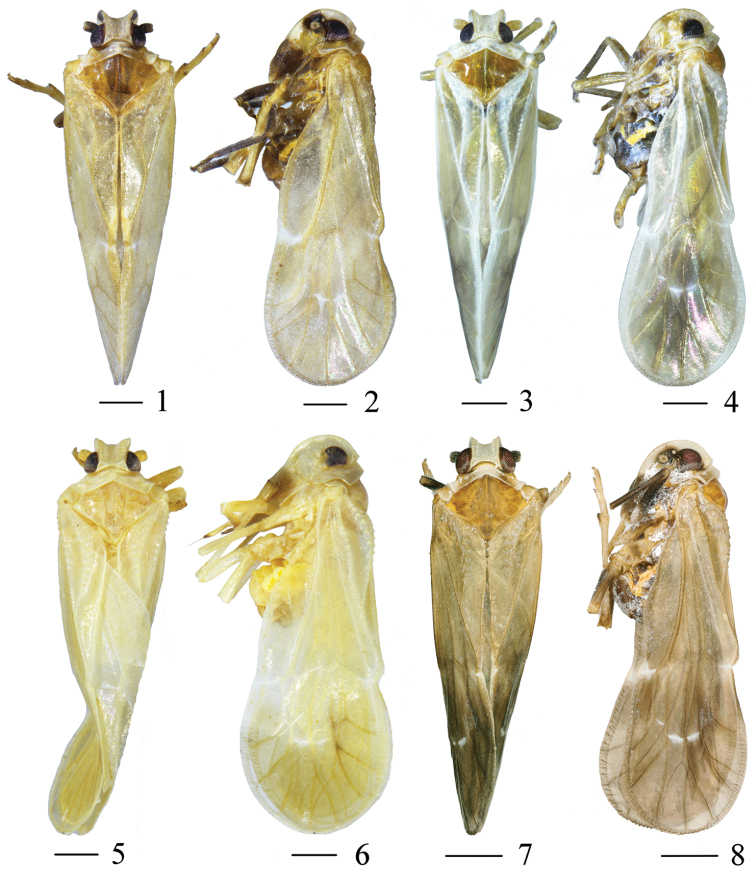
*Metanigrus chromus* sp. nov., male **1** dorsal view **2** lateral view. *Metanigrus guttatus* sp. nov., male **3** dorsal view **4** lateral view. *Metanigrus gremius* sp. nov., male **5** dorsal view **6** lateral view. *Metanigrus spinatus* sp. nov., male **7** dorsal view **8** lateral view. Scale bars: 0.5 mm.

General color grayish yellow (Figs 1, 2). Vertex yellowish white. Eyes blackish red. Frons whitish yellow, a yellowish-brown stripe in middle, with two brownish-black patches on both sides. Clypeus black-brown. Pronotum sublaterally with brownish-black patches. Mesonotum yellowish brown, with brown stripe medially. Legs yellowish brown. Tegmen hyaline, veins brownish. Wings semitransparent. Abdomen brown.

#### Head and thorax.

**Figures 9–19.**
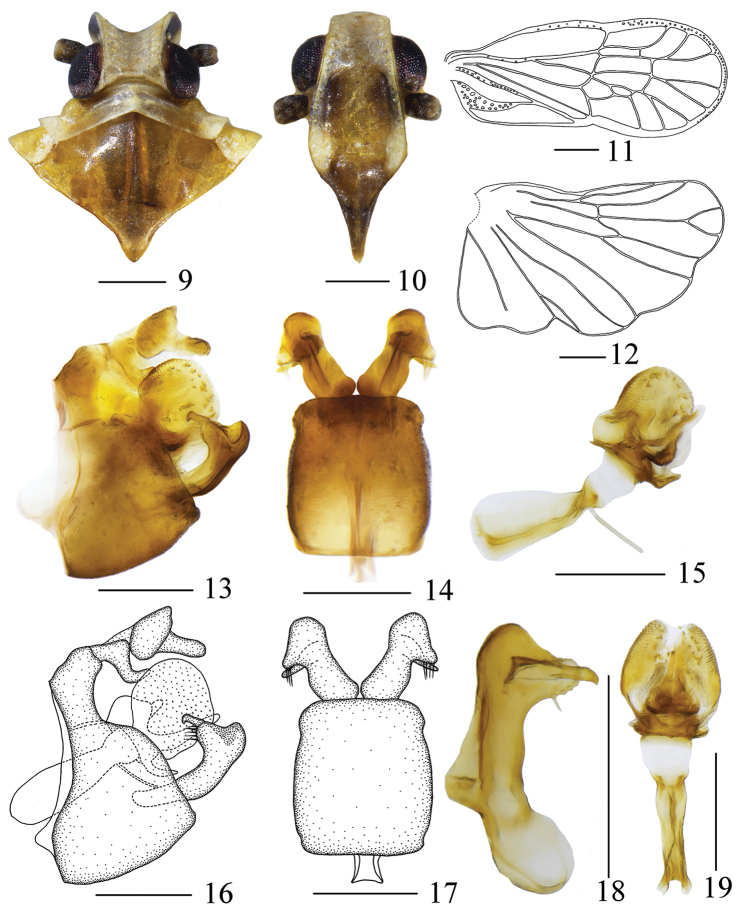
*Metanigrus chromus* sp. nov., male **9** head and thorax, dorsal view **10** frons, ventral view **11** tegmina **12** wing **13, 16** pygofer, lateral view **14, 17** gonostyli and pygofer, ventral view **15** aedeagus, left lateral view **18** gonostyli, dorsal view **19** aedeagus, ventral view. Scale bars: 0.3 mm.

Head (Figs 1, 9) significantly narrower than pronotum, median carina absent. Vertex shorter in middle line than wide at base (1: 1.51), with two posterolateral areolets at base not meeting medially. Frons (Fig. 10) subrectangular, without median carina, longer in middle line than wide at widest portion (about 1.45: 1), lateral carinae with a dense row of sensory pits along outer margin. Postclypeus (Fig. 10) with distinct median and lateral carinae. Frontoclypeal suture nearly straight. Rostrum elongate, surpassing hind-coxae. Pronotum (Fig. 9) wider than maximum width of head (including eyes) (1.37: 1), lateral carinae sinuate, median carina present. Mesonotum (Fig. 9) about 6.14 times longer than pronotum in midline, with distinct median carina. Tegmina (Fig. 11) slender, longer than maximal width (2.41: 1), almost as wide at postnodal as prenodal area, veins ScP + R + MP with several sensory pits, MP3 two branched; five postnodal closed cells with C1 smaller than C2, C5 placed next to C3, C4 distally displaced after the nodal line, im, r-m and anterior two-thirds of margin with white areas. Hind wing (Fig. 12) with lateral margin notched at CuA1, CuP, and A2, more strongly at A1; A2 not reaching posterior margin. Metatibiotarsal formula: (3+5)-6-5.

#### Male genitalia.

Pygofer (Figs 13, 14, 16, 17) symmetrical, gradually narrowed towards apex, with sinuate anterior and posterior margins. Pygopher with internal sulcus and almost parallel lateral margins in ventral view. Anal tube (Figs 13, 16) in lateral view, hand-shaped, anal style stout, lateral lobes distally shorter than anal style, basolateral side with a long finger-like process. Aedeagus (Figs 13, 15, 16, 19) acuminate apically, with tubular periandrium, lateral lobes swelling on both sides, surface beset with scale-like productions, ventral lobe slender with an acute apex, dorsal lobes with an acute and a nearly rectangular process; in ventral view, base wider. Gonostyli (Figs 13, 14, 16–18) divided into two parts, inner process curved apically, tapering into a process; in lateral view, outer process convex, bifurcated nearly equally at apex, inner margin with long setae; in ventral view, divergent, duck-shaped.

#### Type material.

***Holotype*:** ♂, China: Hubei, Dabieshan National Natural Reserve (31°03'N, 115°40'E), 2 July 2014, Jian-Kun Long; Paratypes: 2♂♂, 7♀♀, Hubei, Dabieshan National Natural Reserve, 29–30 June 2014, Zhi-Min Chang and Mei-Na Guo; 15♂♂, 24♀♀, Hubei, Dabieshan National Natural Reserve, 2–3 July 2014, Jian-Kun Long, Zheng-Xiang Zhou, Ying-Jian Wang, Mei-Na Guo and Hai-Yan Sun.

#### Etymology.

The species name is derived from the Latin adjective “*chromus*”, referring to frons with two brownish-black patches on both sides.

#### Remarks.

This species is similar to *Metanigrus yami* Tsaur, Yang & Wilson, 1986, but differs from the latter in: (1) frons with two brownish-black patches on both sides (frons without two brownish-black patches on both sides in *M. yami*); (2) basolateral side of anal tube with a long curved finger-like process (basolateral side of anal tube with a short finger-like process in *M. yami*); (3) outer process of gonostyli without a thumb-like process (outer process of gonostyli with a thumb-like process in *M. yami*).

#### Distribution.

China (Hubei) (Fig. 61).

### Metanigrus guttatus

Taxon classificationAnimaliaHemipteraMeenoplidae

Lv & Chen sp. nov.

80A23828-DAEB-5FA5-B072-BC1C0004C1D8

http://zoobank.org/282F525A-00BB-4C2C-AD1B-61726291F562

Figs 3 , 4 , 20–30

#### Measurements.

Total length: male 3.96–4.31 mm (*N* = 13), female 4.44–4.72 mm (*N* = 8).

#### Diagnosis.

The salient features of the new species include: the distal part of tegmina (Fig. 22) with a large black patch; dorsolateral part of anal tube (Figs 24, 27) with a stout finger-like process, a protrusion on the basolateral finger-like process; gonostyli (Figs 24, 27, 29) slender, inner process very narrow, apex of outer process nearly fan-shaped in lateral view.

#### Coloration.

General color yellowish white (Figs 3, 4). Frons whitish yellow. Clypeus black-brown. Vertex and pronotum white. Mesonotum brown. Eyes black. Legs yellowish brown. Tegmina semitransparent, with black patch near end. Wings hyaline. Abdomen brown.

#### Head and thorax.

**Figures 20–30.**
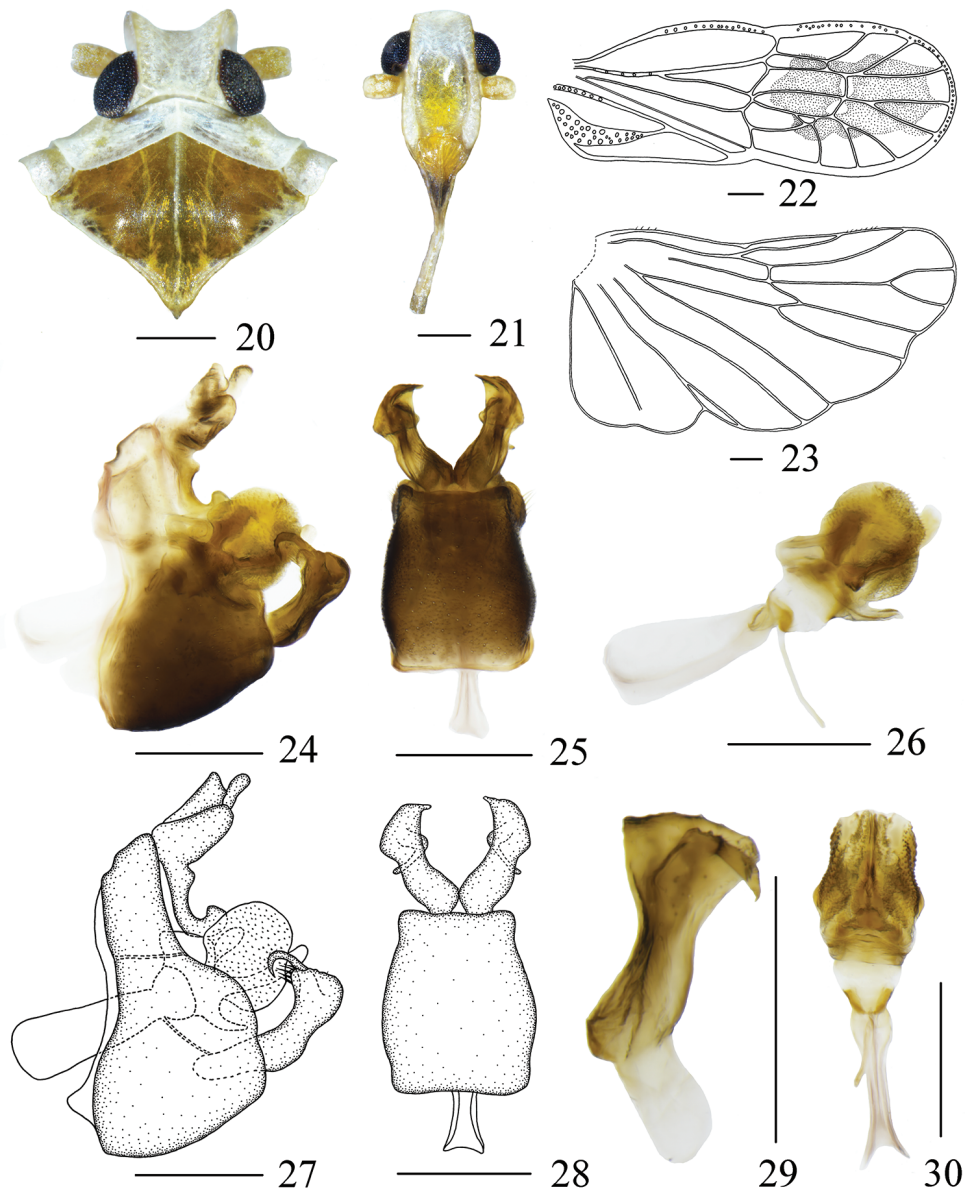
*Metanigrus guttatus* sp. nov. , male **20** head and thorax, dorsal view **21** frons, ventral view **22** tegmina **23** Wing **24, 27** pygofer, lateral view **25, 28** gonostyli and pygofer, ventral view **26** aedeagus, left lateral view **29** gonostyli, dorsal view **30** aedeagus, ventral view. Scale bars: 0.3 mm.

Head (Figs 3, 20) significantly narrower than pronotum, without median carina. Vertex shorter in middle line than wide at base (1: 1.77), with two posterolateral areolets at base not meeting medially. Frons (Fig. 21) subrectangular, without median carina, longer in middle line than wide at widest portion (about 1.67: 1), lateral carinae with a dense row of sensory pits along outer margin. Postclypeus (Fig. 21) with distinct median and lateral carinae. Frontoclypeal suture nearly straight. Rostrum elongate, in repose well surpassing hind-coxae. Pronotum (Fig. 20) wider than maximum width of head (including eyes) (1.71: 1), with median carina and sinuate lateral carinae. Mesonotum (Fig. 20) about 4.57 times longer than pronotum in midline, with distinct median carina. Tegmina (Fig. 22) slender, longer than maximal width (2.59: 1), almost as wide at postnodal as prenodal area, veins ScP + R + MP with several sensory pits, MP3 two branched; five postnodal closed cells with C1 smaller than C2, C5 placed next to C3, C4 distally displaced after the nodal line, im, r-m and anterior two-thirds of margin with white areas. Hind wing (Fig. 23) with lateral margin notched at CuA1, CuP, and A2, more strongly at A1; A2 not reaching posterior margin. Metatibiotarsal formula: (3+5)-6-5.

#### Male genitalia.

Pygofer (Figs 24, 25, 27, 28) symmetrical, gradually narrowed towards apex, with sinuate anterior and posterior margins. Pygopher with internal sulcus, with slightly curved lateral margins in ventral view. Anal tube (Figs 24, 27) in lateral view, hand-shaped, anal style slender, lateral lobes distally shorter than anal style, dorsolateral part with a stout finger-like process, a protrusion on the basolateral finger-like process. Aedeagus (Figs 24, 26, 27, 30) acuminate apically, with tubular periandrium, lateral lobes swelling on both sides, surface beset with scale-like productions, ventral lobe slender with acute apex, dorsal lobes with an acute and a nearly rectangular process; in ventral view, base wider. Gonostyli (Figs 24, 25, 27–29) slender, divided into two parts, inner process very narrow, curved apically, tapering into a process; in lateral view, outer process convex, apex nearly fan-shaped, inner margin with long setae; in ventral view, divergent, top of outer gonostyli with a pointed needle-like structure on the inner side.

#### Type material.

***Holotype*:** ♂, China: Guizhou, Zhijin county (26°35'N, 105°53'E), 18 July 2020, Sha-Sha Lv; Paratypes: 12♂♂, Guizhou, Zhijin county, 18 July 2020, Sha-Sha Lv, Feng-E Li, Xiao-Ya Wang and Nian Gong; 8♀♀, Guizhou, Zhijin county, 18 July 2020, Sha-Sha Lv, Feng-E Li and Nian Gong.

#### Etymology.

The species name is derived from the Latin adjective “*guttatus*”, referring to the tegmina with a patch.

#### Remarks.

This species is similar to *Metanigrus chromus* but differs from the latter in: (1) distal part of tegmina with a large black patch (distal part of tegmina without a large black patch in *M. chromus*); (2) frons without two brownish-black patches on both sides (frons with two brownish-black patches on both sides in *M. chromus*); (3) dorsolateral part of anal tube with a finger-like process (dorsolateral part of anal tube without a finger-like process in *M. chromus*).

#### Distribution.

China (Guizhou) (Fig. 61).

### Metanigrus gremius

Taxon classificationAnimaliaHemipteraMeenoplidae

Lv & Chen sp. nov.

D2B1F0CF-0969-59AF-8CB2-6D440CDADF31

http://zoobank.org/8E2251AE-C6CC-40B5-879E-06FCC3B8DFC9

Figs 5 , 6 , 31–41

#### Measurements.

Total length: male 3.0–3.5 mm (*N* = 4), female 5.1 mm (*N* = 1).

#### Diagnosis.

The salient features of the new species include: general color (Figs 5, 6) yellowish white; dorsolateral and middle parts of anal tube (Figs 35, 38) each with a finger-like process; outer process of gonostyli (Figs 35, 38) convex and arcuate with nearly square process at apex in lateral view.

#### Coloration.

General color yellowish white (Figs 5, 6). Mesonotum light brown. Eyes black. Legs yellowish brown. Tegmina semitransparent. Wings hyaline. Abdomen yellowish white.

#### Head and thorax.

**Figures 31–41.**
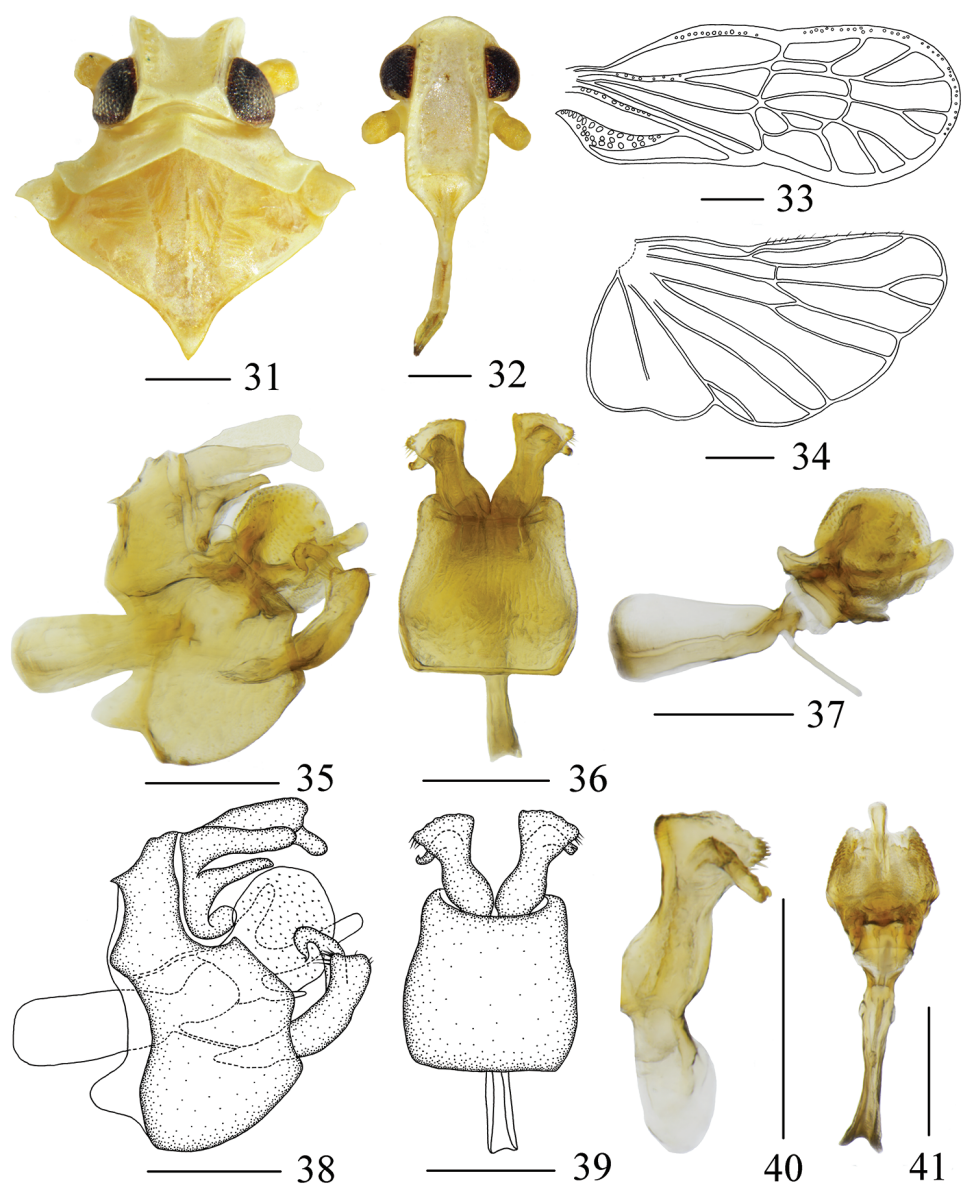
*Metanigrus gremius* sp. nov. , male **31** head and thorax, dorsal view **32** frons, ventral view **33** tegmina **34** wing **35, 38** pygofer, lateral view **36, 39** gonostyli and pygofer, ventral view **37** aedeagus, left lateral view **40** gonostyli, dorsal view **41** aedeagus, ventral view. Scale bars: 0.3 mm.

Head (Figs 5, 31) significantly narrower than pronotum, without median carina. Vertex shorter in middle line than wide at base (1: 1.99), with two posterolateral areolets at base not meeting medially. Frons (Fig. 32) subrectangular, without median carina, longer in middle line than wide at widest portion (about 1.93: 1), lateral carinae with a dense row of sensory pits along outer margin. Postclypeus (Fig. 32) with distinct median and lateral carinae. Frontoclypeal suture nearly straight. Rostrum elongate, surpassing hind-coxae. Pronotum (Fig. 31) wider than maximum width of head (including eyes) (1.43: 1), with median carina and sinuate lateral carinae. Mesonotum (Fig. 31) about 5.96 times longer than pronotum in midline, with distinct median carina. Tegmina (Fig. 33) slender, longer than maximal width (2.59: 1), almost as wide at postnodal as prenodal area, veins ScP + R + MP with several sensory pits, MP3 two branched; five postnodal closed cells with C1 smaller than C2, C5 placed next to C3, C4 distally displaced after the nodal line, im, r-m and anterior two-thirds of margin with white areas. Hind wing (Fig. 34) with lateral margin notched at CuA1, CuP, and A2, more strongly at A1; A2 not reaching posterior margin. Metatibiotarsal formula: (3+5)-6-5.

#### Male genitalia.

Pygofer (Figs 35, 36, 38, 39) symmetrical, gradually narrowed towards the apex, with sinuate anterior and posterior margins. Pygopher with internal sulcus and slightly curved lateral margins in ventral view. Anal tube (Figs 35, 38) in lateral view, hand-shaped, anal style slender, lateral lobes distally shorter than anal style, dorsolateral and middle parts each with a finger-like process, basolateral side with a finger-like process. Aedeagus (Figs 35, 37, 38, 41) acuminate apically, with tubular periandrium, lateral lobes swelling on both sides, surface beset with scale-like productions, ventral lobe slender with acute apex, dorsal lobes with a short finger-like process; in ventral view, base wider. Gonostyli (Figs 35, 36, 38–40) divided into two parts, inner process curved apically, tapering into a process; in lateral view, outer process convex and arcuate with nearly square process at apex; in ventral view, divergent, inner part beak-shaped backward, apex of outer process nearly fan-shaped.

#### Type material.

***Holotype*:** ♂, China: Yunnan, Daweishan National Natural Reserve (22°94'N, 103°70'E), 4 June 2016, Ying-Jian Wang; Paratypes: 3♂♂, Yunnan, Daweishan National Natural Reserve, 4 June 2016, Ying-Jian Wang; 1♀, Yunnan, Daweishan National Natural Reserve, 4 June 2016, Qiang Luo.

#### Etymology.

The species name is derived from the Latin adjective “*gremius*”, referring to general yellowish white.

#### Remarks.

This species is similar to *Metanigrus guttatus* but differs from the latter in: (1) distal part of tegmina without large, black patch (distal part of tegmina with large, black patch in *M. guttatus*); (2) dorsolateral and middle parts of anal tube each with a finger-like process (only dorsolateral part of anal tube with a finger-like process in *M. guttatus*); (3) outer process of gonostyli nearly square at apex in lateral view (outer process of gonostyli nearly fan-shaped at apex in lateral view in *M. guttatus*).

#### Distribution.

China (Yunnan) (Fig. 61).

### Metanigrus spinatus

Taxon classificationAnimaliaHemipteraMeenoplidae

Lv, Chen & Bourgoin sp. nov.

04AB8694-867E-5902-8270-A4BF0ABF6527

http://zoobank.org/BF15FE85-4D07-4789-B214-C2A663A21883

Figs 7 , 8 , 42–52

Metanigrus yami Liu & Qin 2020: 27–29 (misidentification)

#### Measurements.

Total length: male 3.01–3.52 mm (*N* = 18), female 3.68–4.13 mm (*N* = 15).

#### Diagnosis.

The salient features of the new species include: frons (Fig. 43) with a big, longitudinal and black brown marking in middle; middle part of anal tube (Figs 46, 49) with a finger-like process, a protrusion on the basolateral finger-like process; inner margin of outer gonostyli (Figs 46, 49) strongly protruding, outer margin gentle, bifurcated unequally in lateral view.

#### Coloration.

General color fuscous (Figs 7, 8). Vertex and pronotum yellowish white. Eyes reddish black. Frons yellowish white, with a big, longitudinal, black-brown marking in middle, reaching apex of frons. Mesonotum ocherous. Legs yellowish brown. Tegmina semitransparent, veins light yellow. Wings hyaline. Abdomen brown.

#### Head and thorax.

**Figures 42–52.**
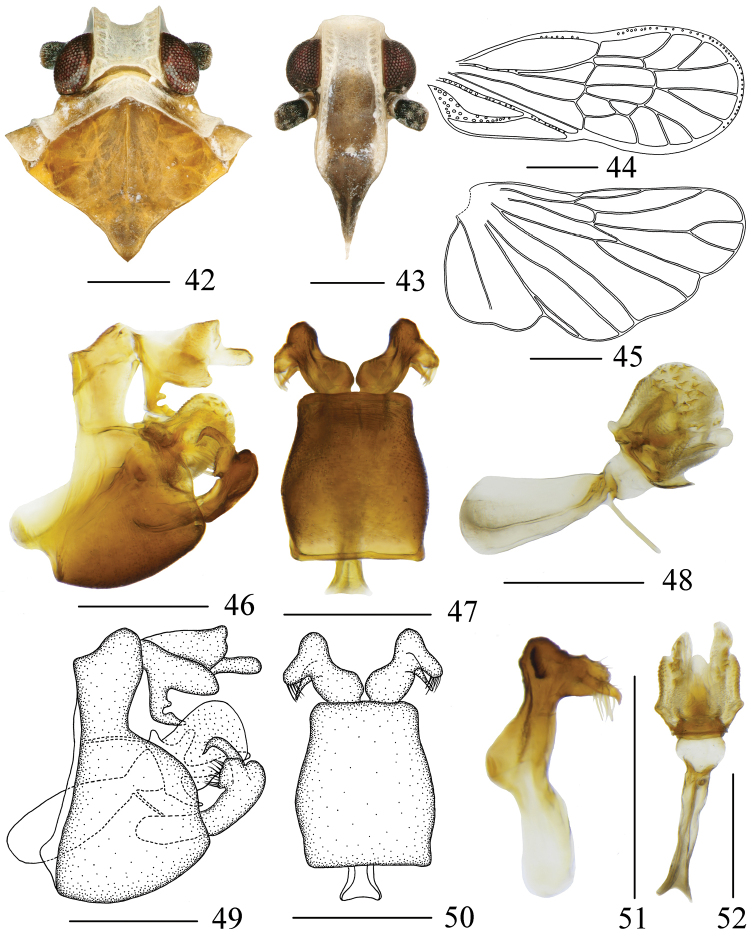
*Metanigrus spinatus* sp. nov. , male **42** head and thorax, dorsal view **43** frons, ventral view **44** tegmina **45** wing **46, 49** pygofer, lateral view **47, 50** gonostyli and pygofer, ventral view **48** aedeagus, left lateral view **51** gonostyli, dorsal view **52** aedeagus, ventral view. Scale bars: 0.3 mm.

Head (Figs 7, 42) significantly narrower than pronotum, without median carina. Vertex (Fig. 42) shorter in middle line than wide basally (1: 1.84), with two posterolateral areolets at base not meeting medially. Frons (Fig. 43) subrectangular, without median carina, longer in middle line than wide at widest portion (about 1.86: 1), lateral carinae with a dense row of sensory pits along outer margin. Postclypeus (Fig. 43) with distinct median and lateral carinae. Frontoclypeal suture nearly straight. Rostrum elongate, in repose well surpassing hind-coxae. Pronotum (Fig. 42) wider than maximum width of head (including eyes) (1.35: 1), with median carina and sinuate lateral carinae. Mesonotum (Fig. 42) about 5.62 times longer than pronotum in midline, with distinct median carina. Tegmina (Fig. 44) slender, longer than maximal width (2.45: 1), almost as wide at postnodal as prenodal area, veins ScP + R + MP with several sensory pits, MP3 two branched; five postnodal closed cells with C1 smaller than C2, C5 placed next to C3, C4 distally displaced after the nodal line, im, r-m and anterior two-thirds of margin with white areas. Hind wing (Fig. 45) with lateral margin notched at CuA1, CuP, and A2, more strongly at A1; A2 not reaching posterior margin. Metatibiotarsal formula: (3+5)-6-5.

#### Male genitalia.

Pygofer (Figs 46, 47, 49, 50) symmetrical, gradually narrowed towards apex, with sinuate anterior and posterior margins. Pygopher with internal sulcus and slightly curved lateral margins in ventral view. Anal tube (Figs 46, 49) in lateral view, hand-shaped, anal style long, lateral lobes distally shorter than anal style, middle part with a finger-like process, a protrusion on the basolateral finger-like process. Aedeagus (Figs 46, 48, 49, 52) acuminate apically, with tubular periandrium, lateral lobes swelling on both sides, surface beset with scale-like productions, ventral lobe slender with acute apex, dorsal lobe with a little finger-like process; in ventral view, base wider. Gonostyli (Figs 46, 47, 49–51) divided into two parts, inner process curved apically, tapering into a process; in lateral view, inner margin of outer process strongly protruding, outer margin gentle, bifurcated unequally; in ventral view, divergent, outer process hammer-shaped.

#### Type material.

***Holotype*:** ♂, China: Menglun, Yunnan Province (21°93'N, 101°26'E), 21 June 2019, Feng-E Li; Paratypes: 17♂♂15♀♀, Menglun, Yunnan Province, 22 June 2019, Feng-E Li, Yan Zhi and Nian Gong.

#### Etymology.

The species name is derived from the Latin adjective “*spinatus*”, referring to the middle part of anal tube with a finger-like process.

#### Remarks.

This species is similar to *Metanigrus chromus* but differs from the latter in: (1) frons with a big, longitudinal and black brown marking in middle (frons with two brownish-black patches on both sides in *M. chromus*); (2) middle part of anal tube with a finger-like process (middle part of anal tube without a finger-like process in *M. chromus*); (3) outer process of gonostyli bifurcated unequally at apex in lateral view (outer process of gonostyli bifurcated nearly equal at apex in lateral view in *M. chromus*).

#### Distribution.

China (Yunnan) (Fig. 61).

## Discussion

**Figures 53–55.**
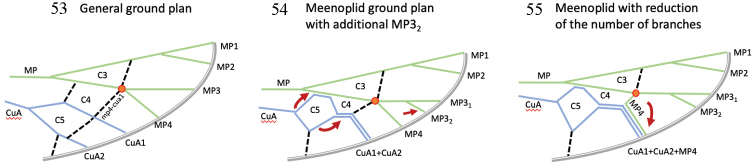
Interpretation of Meenoplidae tegmina pattern. **53** general planthopper pattern (from Bourgoin et al. 2015) **54** general Meenoplidae pattern (modified from Bourgoin 1987) with additional late forking of MP3 into two terminals (MP31 and MP32) **55**Meenoplidae with reduced number of terminal veins: MP3 remains forked, MP4 forks as usual and merged posteriorly with CuA1+CuA2.

From a general planthopper tegmina pattern (Bourgoin et al. 2015), the ground plan tegmina pattern in meenoplids (Fig. 53; Bourgoin 1997) shows an MP vein ending with 4 terminals with MP3 separating from MP4 at the subnodal line, CuA1 merging distally with CuA2, C5 and C4 placed in parallel with C4 distally closed by a short mp4-cua1 veinlet part of the subnodal line. In a few genera MP3 might fork later after the subnodal line into MP3-1 and MP3-2 (*Anigrus* Stål, 1866; *Caledonisia* Bourgoin, 1987; *Muirisinia* Bourgoin, 1987) (Fig. 54). During this study we observed an important variability in the tegmina venation in two of species described: *M. gremius* sp. nov. and *M. spinatus* sp. nov., which allow us to provide some more precisions about the tegmina venation in meenoplids.

*Metanigrus*, as described by Tsaur et al. (1986), fits perfectly well with the meenoplid tegmina ground plan, with C4 almost in series with C5 and the forking of MP3+4 at the subnodal line. However, all the new species described here differ in having five terminal branches with a late forking of the corresponding MP3 branch, distinctly well after the subnodal line (Fig. 55). Accordingly, two interpretations are therefore possible: (1) this late forking corresponds to a late fork of MP3+4 into MP3 and MP4, or (2) this late forking is a second division of MP3, as known to occur is some other meenoplid genera. Accordingly, MP4 would separate earlier at the subnodal line and join CuA1+CuA2 with which it remains merged. The recent description of *M. rotundatus* by Liu and Qin (2020) fits the intermediate state of five MP branches, and the variability of the tegmina pattern observed in this study allows us to choose the second interpretation.

**Figures 56–60.**
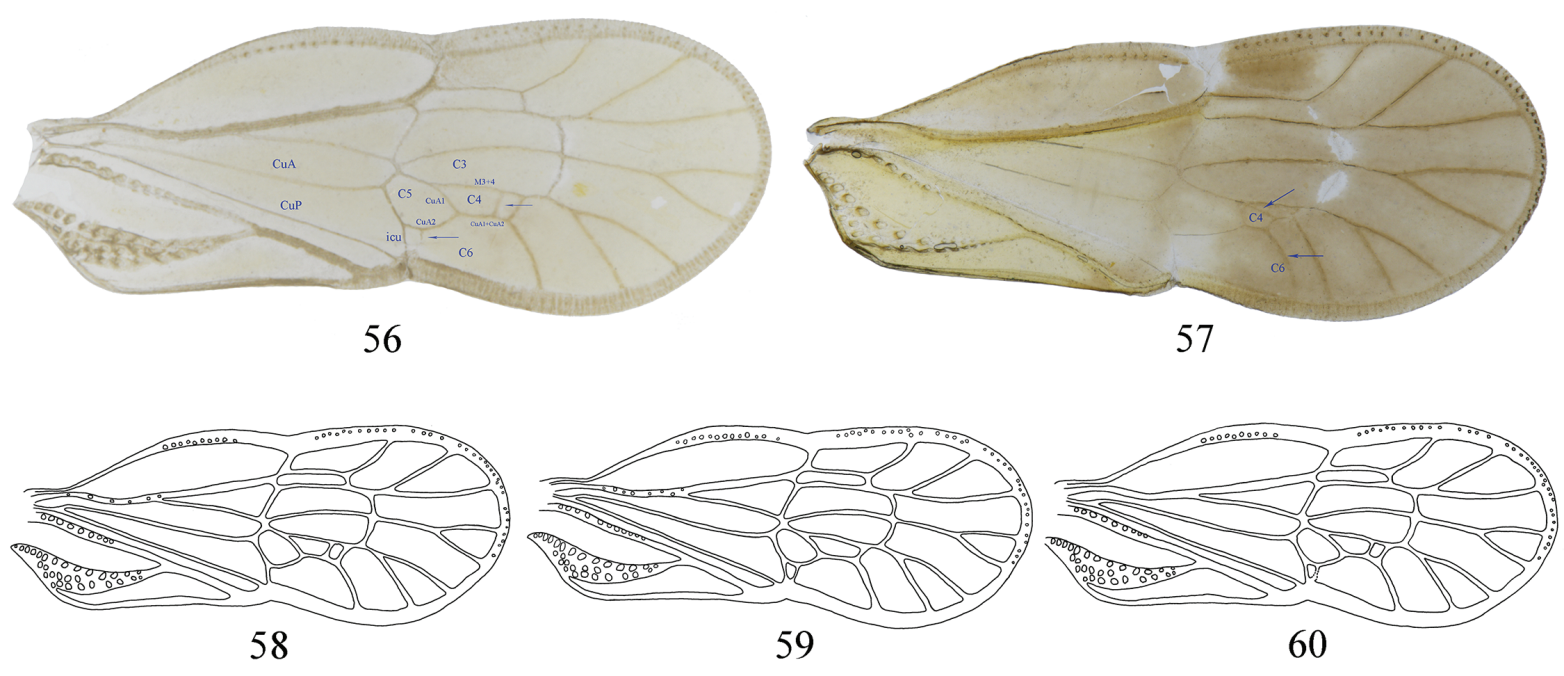
Tegmina. **56, 58–60***Metanigrus gremius* sp. nov. **57***Metanigrus spinatus* sp. nov.

In *M. gremius* sp. nov. several additional veinlets might occur, include a small one doubling icu and/or another one doubling mp4-cua1 (Figs 56, 58–60). In *M. spinatus* sp. nov. (Fig. 57), we observed another type of variation with a pattern with six terminals, as in *M. rotundatus*. Only one-fifth of the specimens possessed these characteristics. An interpretation of a late forking of MP3+4 would lead us to also consider a return to a distally separated CuA1 and CuA2, and we prefer the most parsimonious modification, a normal forking of MP3+4, a normal separation of MP4 from CuA1+CuA2, and just a 2-branched MP3. This observation led us to consider that the 5-branched MP is not a diagnostic character for *M. rotundatus*, as used in the key to species (Liu and Qin 2020), but just punctual variation; this species’ very distinctive gonostyli is a good character for distinguishing this species without difficulty.

**Figure 61.**
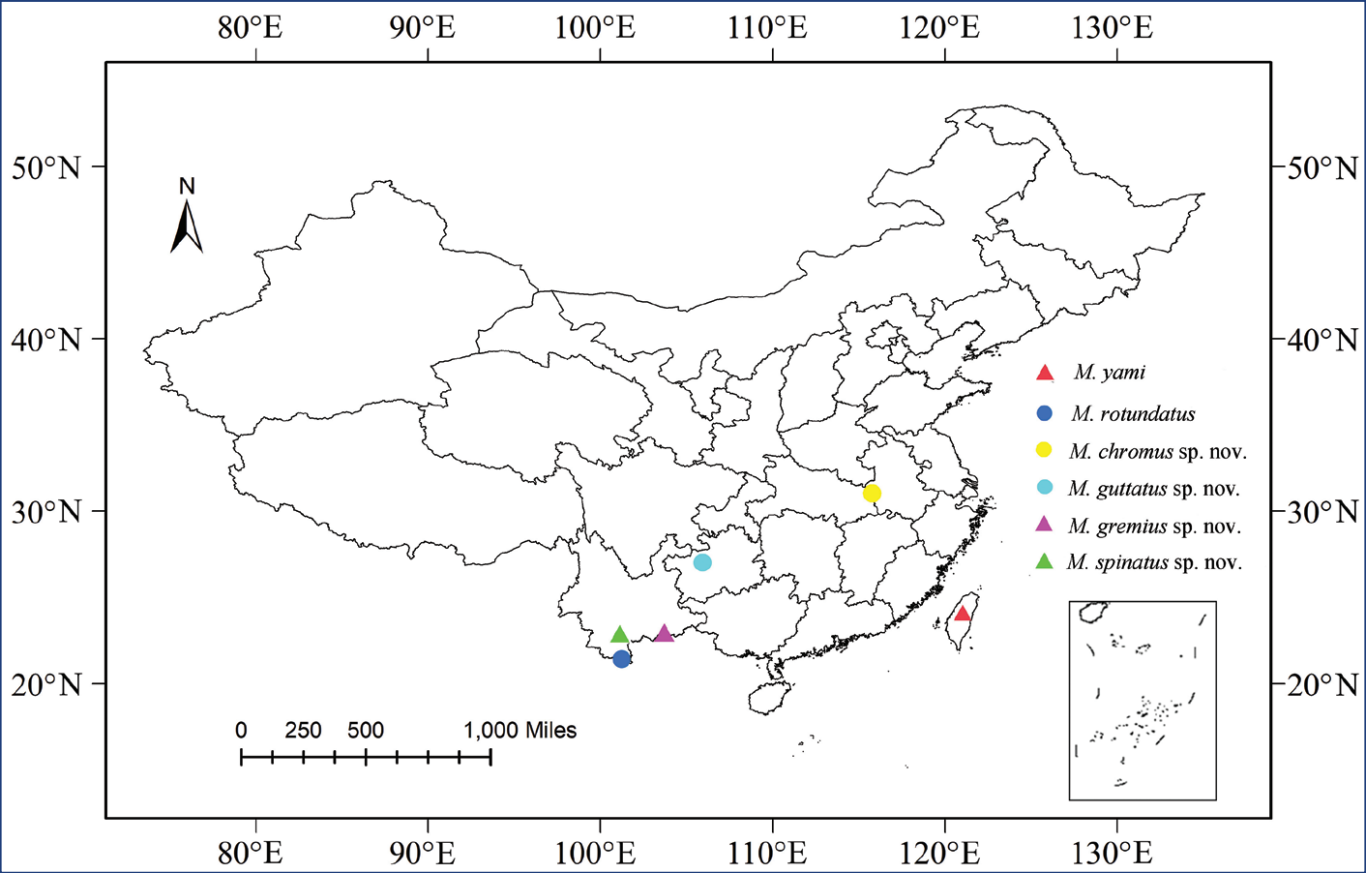
Geographic distributions of *Metanigrus* species.

According to the geographic distribution (Fig. 61) of the genus *Metanigrus*, all species are distributed in the Oriental realm (Holt et al. 2013) and especially in Yunnan in southwestern China. Excluding the invasive alien species, such a distribution fits well with the general distribution currently known for meenoplidae taxa (Bourgoin 2021). If the group probably did not extent further north in China into the Tibetian and Sino-Japanese realms (Holt et al. 2013), we would expect more new species to be discovered in China from the already taxonomically richer Oriental realm. The genus might be also widely distributed to the south on the Indochinese Peninsula. However, the genus currently remains endemic to China.

## Supplementary Material

XML Treatment for Metanigrus

XML Treatment for Metanigrus chromus

XML Treatment for Metanigrus guttatus

XML Treatment for Metanigrus gremius

XML Treatment for Metanigrus spinatus
